# Personality traits and their clinical associations in trichotillomania and skin picking disorder

**DOI:** 10.1186/s12888-021-03209-y

**Published:** 2021-04-21

**Authors:** Jon E. Grant, Samuel R. Chamberlain

**Affiliations:** 1 Department of Psychiatry & Behavioral Neuroscience, University of Chicago, 5841 S. Maryland Avenue, MC 3077, Chicago, IL 60637 USA; 2 Department of Psychiatry, Faculty of Medicine, University of Southampton, Southampton, UK; 3 Southern Health NHS Foundation Trust, Southampton, UK

**Keywords:** Trichotillomania, Skin picking, Personality, Traits

## Abstract

**Background:**

Despite being discussed in the psychiatric literature for decades, very little is known about personality features associated with trichotillomania and skin picking disorder (known as body focused repetitive behavior disorders, BFRBs); and the contribution of personality traits to their clinical presentations.

**Aim:**

The present study assessed personality traits in a large and well-characterized sample of adults with either trichotillomania or skin picking disorder or both.

**Methods:**

Adults (*n* = 98, aged 18–65 years), with trichotillomania (*n* = 37), skin picking disorder (*n* = 32), both trichotillomania and skin picking disorder (*n* = 10), and controls (*n* = 19) were enrolled. Participants completed self-report questionnaires to quantify personality (NEO Personality Inventory), as well as extent/severity of picking/pulling symptoms, mood and anxiety, impulsive and perfectionistic tendencies, and neurocognitive functioning. Group differences were characterized and correlations with other measures were examined.

**Results:**

In comparison to controls, BFRBs had elevated neuroticism scores (*p* < 0.001), lower extraversion scores (*p* = 0.023), and lower conscientiousness scores (*p* = 0.007). Neuroticism was significantly related to both hair pulling (*r* = 0.24, *p* < 0.001) and skin picking severity (*r* = 0.48, *p* < 0.001), as well as elevated perceived stress, worse anxiety and depressive symptoms, and poorer quality of life. Introversion (i.e. lower extraversion) was significantly associated with worse picking severity, higher perceived stress, and higher depression. Lack of conscientiousness was significantly associated with more depression, impulsivity, and perceived stress.

**Discussion:**

Personality traits of neuroticism, introversion, and lack of conscientiousness are heightened in individuals with BFRBs and show strong associations with a number of clinically relevant features of illness. The holistic understanding and treatment of these disorders is likely to require consideration of dimensional traits such as these.

## Introduction

Trichotillomania and skin picking disorder are characterized, respectively, by recurrent pulling and picking, resulting in hair loss or skin excoriations, as well as functional impairment or distress [1]. Understanding factors that may contribute to the picking or pulling behaviors, or even assist with coping with the behaviors, may be valuable as these personality traits may add to our understanding of these disorders and contribute to the development of more effective treatments. Unfortunately, even though research on these under-recognized disorders is increasing, personality traits in people with trichotillomania and skin picking disorder have received little investigation.

Only a few studies to date have examined personality features of people with these disorders. In an early study, Stanley and colleagues [2] assessed personality traits in people with trichotillomania (*n* = 8) and obsessive compulsive disorder (OCD) (*n* = 13), and found that adults with trichotillomania were more extraverted than OCD patients (using the Eysenk Personality Questionnaire). Using the Temperament and Character Inventory, Lochner and colleagues [3] assessed 54 participants with trichotillomania and 278 with OCD and found that those with trichotillomania scored significantly higher on novelty seeking, whereas those with OCD reported significantly greater harm avoidance. In one of the few studies of skin picking disorder (*n* = 21), Lochner and colleagues [4] found that people with skin picking disorder scored high (compared to published normative data) on measures of reward dependence and harm avoidance, but not on novelty-seeking, using the Tridimensional Personality Questionnaire. In a study of 43 adults with trichotillomania and 43 controls using the NEO-Fave Factor Inventory, Hagh-Shenas and colleagues found that trichotillomania was associated with significantly higher scores on all neuroticism subscales and significantly lower scores on the compliance sub-scale of Agreeableness [5]. Finally, Keuthen and colleagues [6] examined personality factors in 164 adults with trichotillomania using the NEO-Five Factor Inventory and found that elevated openness, neuroticism, and less agreeableness were all associated with both greater pulling severity and that higher neuroticism was associated with less pulling control.

Reports on personality characteristics of trichotillomania and skin picking disorder have been few in number and have focused primarily on trichotillomania. The present study assessed personality traits in a large and well-characterized sample of adults with either trichotillomania or skin picking disorder or both. Based on the literature and our clinical experience, we hypothesized that people with trichotillomania or skin picking disorder would have high neuroticism scores and that higher scores would correlate with greater symptom severity.

## Methods

### Participants

Non-treatment seeking adults (*n* = 98), ages 18–65 with a current Diagnostic and Statistical Manual Version 5 (DSM-5) diagnosis of trichotillomania (*n* = 37), skin picking disorder (*n* = 32), both trichotillomania and skin picking disorder (*n* = 10), and controls (*n* = 19) were enrolled. Participants with trichotillomania, skin picking disorder, or both conditions, were grouped together as having a body-focused repetitive behavior (BFRB). Participants were recruited from March 2017 to September 2018 using online advertisements and referrals. Exclusion criteria included: change in psychotropic medication or dose 3 months prior to study entry, or any medical condition that would preclude completion of questionnaires. Additional exclusion criteria for controls was whether they had any current or lifetime psychiatric disorder. All participants completed the Mini International Neuropsychiatric Interview 7.0 (MINI 7.0), a clinician-administered interview, to assess for comorbid conditions [7].

### Assessments

All BFRB and control participants completed the NEO-Five-Factor Inventory (NEO-FFI) [8]. The NEO-FFI is a reliable and valid 60-item self-report measure that assesses the following personality domains: Neuroticism, Extraversion, Openness, Agreeableness, and Conscientiousness. Items are rated on a five-point Likert scale, with higher scores indicating a greater degree of each of the domains.

Additionally, participants completed a semi-structured interview to acquire information on demographics and on the clinical characteristics of BFRBs. Other measures included the following, which were all self-report questionnaires: Massachusetts General Hospital Hair Pulling Scale (MGH-HPS) [9], a severity scale of hair pulling; Skin Picking Scale-Revised (SPS-R) [10], a scale of picking severity; the Perceived Stress Scale (PSS) [11]; the Barratt Impulsivity Scale (BIS-11, total score) [12]; Sheehan Disability Scale (SDS) [13]; Short Mood and Feelings Questionnaire (dimensional measure of depression) [14]; Quality of Life Inventory (QOLI) [15]; Frost Multidimensional Perfectionism Scale (FMPS) [16]; and the adult version of the Screen for Child Anxiety and Related Disorders (dimensional measure of anxiety) [17]. The reason for including these scales was that we wished to characterize presence and severity of trichotillomania and picking symptoms, as well as to quantify variables implicated in the manifestation of these and related conditions (i.e. impulsivity and rigidity) [18, 19], as well as dimensional measures of depression and anxiety.

In addition to paper-pencil measures, each participant underwent neurocognitive testing, using tasks from the Cambridge Neurocognitive Test Automated Battery (CANTAB; http://www.cantab.com), which were counter-balanced in terms of order of administration. These tasks included: Extra-dimensional set-shifting errors (IED) [20]; stop-signal reaction times (SSRT) (assessing response inhibition) [21]; and the Cambridge Gamble Task (CGT) (assessing decision making) [22]. We included these tasks as the cognitive domains they measure have been implicated in one or more of the obsessive-compulsive related disorders [23–26]. The IED is adapted from the classic Wisconsin Card Sorting Test, and examines the ability of participants to learn and flexibly adapt a learnt ‘rule’ in order to select correct images presented by the computer. The SSRT is a classic test of response inhibition whereby volunteers attempt to withhold a usual button press when a stop-signal occurs (auditory beep) following presentation of a cue. The CGT evaluates decision-making across multiple domains: on each trial, volunteers choose to gamble a particular proportion of their cumulative points on whether a token is hidden behind a red or blue box. Full descriptions of the tests are available on www.cambridgecognition.com, along with a detailed bibliography.

### Data analysis

Total scores on each NEO domain were compared between all people with BFRBs and controls using one-way analysis of variances (ANOVAs), after confirming assumptions were met. Note that one-way ANOVA is algebraically equivalent to standard independent sample t-tests, as applied for two group comparisons. We also then considered, for any significant results, whether people with TTM differed from people with SPD on the given measure, again using ANOVA. For significant results, Cohen’s D effect sizes were reported to contextualize their likely importance (small 0.2–0.49; medium: 0.5–0.69; large: 0.70 or above). We also explored – for these significant measures – relationships between NEO scores and other measures in those with BFRBs using Spearman’s r. Statistical significance was defined as *p* < 0.05 uncorrected for the small number of ANOVAs relating to the NEO total scores (there being strong a priori hypotheses and a small number of tests; and very low power if statistical correction had been undertaken at the intended sample size). For correlations, we employed Bonferroni correction due to the large number of tests and adequate power to do so. Effect sizes for significant correlations were interpreted per convention (*r* = 0.10–0.29: small; 0.30–0.49: medium; 0.50 or higher: large). Statistical analyses were conducted using JMP Pro Software.

## Results

The sample comprised 98 individuals, of whom 37 had TTM, 32 SPD, 10 had both, and 19 were controls. The mean age of the sample was 30.1 (8.4) years, 82 (84.5%) were female, and 75 (76.5%) were of White Caucasian ethnicity. The BFRB group and the control group did not differ significantly in terms of age (mean [SD] age in BFRB group: 29.5 [9.7] years; in controls: 26.2 [5.2]; F = 1.995, *p* = .161), nor gender distributions (N [%] female in the BFRB group: 78 [83.9%]; in controls: 15 [79.0%]; Fisher’s exact test *p* = 0.244). Groups did not differ in terms of racial-ethnic status (68 [78.2%] White-Caucasian in BFRBs; 11 [57.9%] in controls; Likelihood Ratio chi-square = 5.991, *p* = 0.200 using all racial-ethnic data/groupings).

**Fig. 1:**
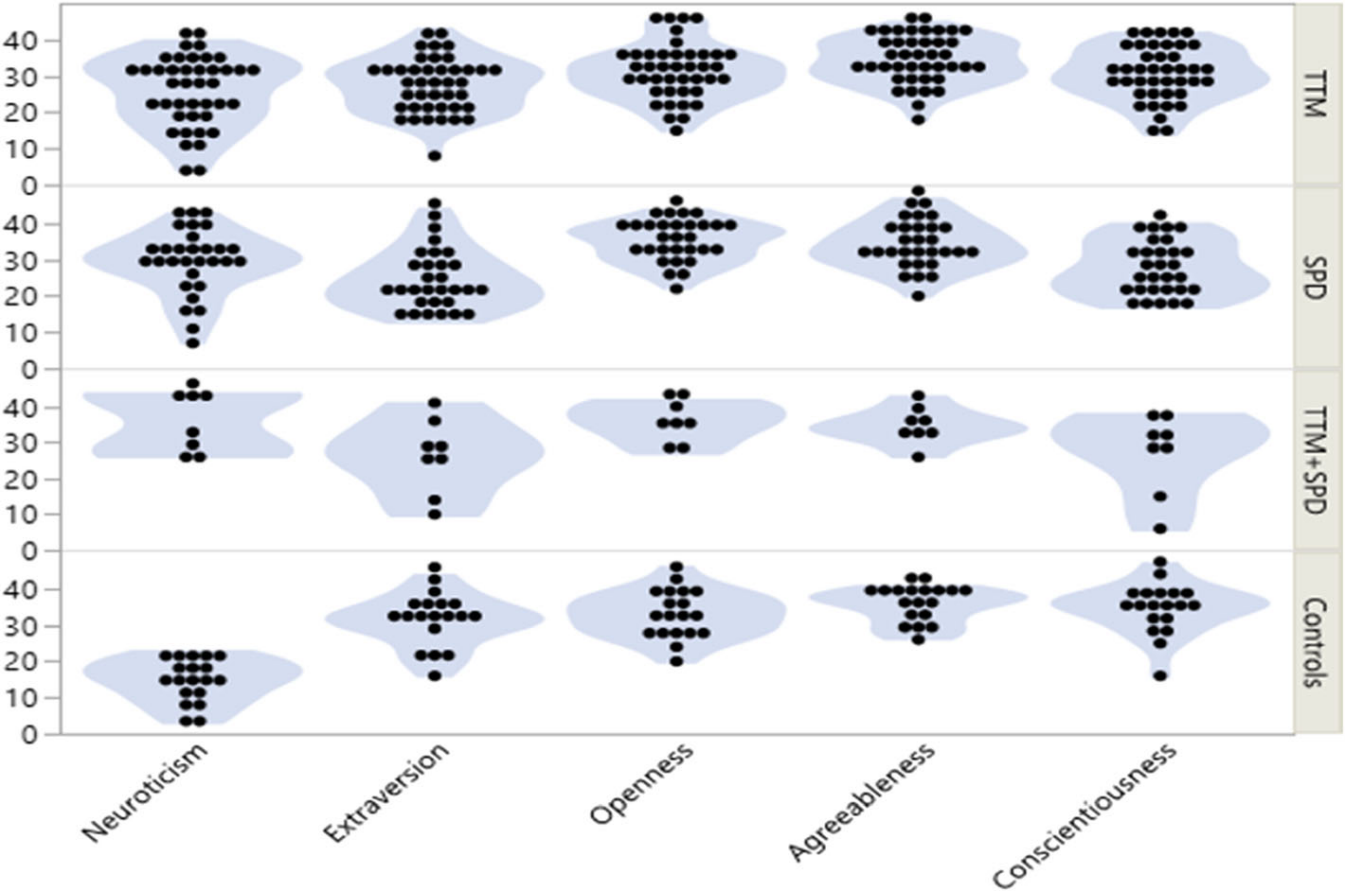
Distributions of total scores on each NEO domain for each sub-group. The graphs show individual data points and violin plots. These are presented for information purposes but our statistical examination did not consider all sub-populations due to limited sample sizes

The total NEO scores for the pooled BFRB group and controls are shown in Table 1 (Fig. 1 shows the scores by sub-groups). As compared to controls, the BFRB group had significantly elevated neuroticism scores (F = 32.85, *p* < 0.001; Cohen’s D = 1.85), lower extraversion scores (i.e. elevated introversion) (F = 5.32, *p* = 0.023; D = 0.59), and lower conscientiousness scores (i.e. elevated lack of conscientiousness) (F = 7.64, *p* = 0.007; D = 0.71). No significant differences between trichotillomania and skin picking disorder cases were evident on these three measures (all ANOVA *p* > 0.10). The BFRB group did not differ on openness scores, nor agreeableness scores, versus controls (both *p* > 0.40).

**Table 1:** NEO-FFI mean (standard deviation) scores in the BFRB group and controls

	BFRBs (***N*** = 79)		Controls (***N*** = 19)	
	Mean	Std Dev	Mean	Std Dev
Neuroticism	27.73	9.50	14.63	5.96
Extraversion	26.15	8.08	30.79	6.92
Openness	32.68	6.87	32.68	6.30
Agreeableness	33.86	6.20	34.89	4.71
Conscientiousness	29.00	7.76	34.37	6.89

Table 2 shows correlations between NEO neuroticism, extraversion, and conscientiousness scores and relevant clinical measures in people with BFRBs. Only findings that were significant at Bonferroni corrected threshold were considered further herein. It can be seen that neuroticism was significantly related to both hair pulling and skin picking severity (small-medium effect sizes) as well as elevated perceived stress (large effect size), worse anxiety and depressive symptoms (large effect sizes), and poorer quality of life (medium effect size). Introversion (i.e. lower extraversion) was significantly associated with worse picking severity, higher perceived stress (large effect size), and higher depression (medium effect sizes). Lack of conscientiousness was significantly associated with more depressive symptoms (medium effect size), higher perceived stress (medium effect size), and higher impulsivity (large effect size).

**Table 2:** Correlations between salient NEO scores and other selected variables in the people with BFRBs. Findings significant after Bonferroni correction (threshold 0.05/42) are shown with an asterisk (*)

Variable	By variable	Spearman Rho	p (uncorrected)	
BIS, total score	Neuroticism	0.25	0.0139	
MGH_HPS	Neuroticism	0.24	< 0.001	*
Perceived Stress	Neuroticism	0.71	<.0001	*
SPS_R	Neuroticism	0.48	<.0001	*
SSRT	Neuroticism	−0.0587	0.6344	
SDS_T	Neuroticism	0.2109	0.0843	
CANTAB_IED	Neuroticism	−0.1532	0.1834	
QOLI_TSCORE	Neuroticism	−0.4189	0.0003	*
MFQ	Neuroticism	0.5568	<.0001	*
FMPS	Neuroticism	0.3458	0.0039	
CGT_RISK_ADJ	Neuroticism	0.0275	0.8125	
CGT_QUAL_DEC_MKG	Neuroticism	−0.0415	0.7198	
CGT_OVERALL_PROP_BET	Neuroticism	0.0198	0.864	
SCARED	Neuroticism	0.6396	<.0001	*
BIS, total score	Extraversion	0.03	0.7904	
MGH_HPS	Extraversion	0	1	
Perceived Stress	Extraversion	−0.4	<.0001	*
SPS_R	Extraversion	−0.35	0.0011	*
SSRT	Extraversion	−0.0615	0.6182	
SDS	Extraversion	−0.1898	0.121	
CANTAB_IED	Extraversion	0.1877	0.1021	
QOLI_TSCORE	Extraversion	0.3419	0.0035	
MFQ	Extraversion	−0.4719	<.0001	*
FMPS	Extraversion	−0.1666	0.1745	
CGT_RISK_ADJ	Extraversion	0.0028	0.9807	
CGT_QUAL_DEC_MKG	Extraversion	0.0072	0.9506	
CGT_OVERALL_PROP_BET	Extraversion	0.0052	0.9644	
SCARED	Extraversion	− 0.3285	0.0118	
BIS, total score	Conscientiousness	−0.56	<.0001	*
MGH_HPS	Conscientiousness	−0.03	0.8	
Perceived Stress	Conscientiousness	−0.4	<.0001	*
SPS_R	Conscientiousness	−0.29	0.0072	
SSRT	Conscientiousness	−0.0683	0.5802	
SDS	Conscientiousness	−0.1337	0.2771	
CANTAB_IED	Conscientiousness	0.1207	0.2958	
QOLI_TSCORE	Conscientiousness	0.3695	0.0015	
MFQ	Conscientiousness	−0.405	0.0006	*
FMPS	Conscientiousness	−0.0392	0.7508	
CGT_RISK_ADJ	Conscientiousness	−0.0847	0.4639	
CGT_QUAL_DEC_MKG	Conscientiousness	−0.0734	0.5257	
CGT_OVERALL_PROP_BET	Conscientiousness	0.0782	0.4989	
SCARED	Conscientiousness	−0.2297	0.0828	

## Discussion

To our knowledge, this is one of the few studies to examine personality features in adults with either trichotillomania or skin picking disorder or both. We also included a control group to contextualize the findings in the patients. Our analyses indicate that increased neuroticism was significantly statistically related to both hair pulling and skin picking severity, as well as anxiety, depression, and perceived stress. Neuroticism conceptually relates to experiencing negative affect (including anxiety/depression), and self-doubting. This is in keeping with a previous study [6] in trichotillomania and extends those findings to skin picking disorder as well. Unlike previous studies [5, 6], however, neither openness nor agreeableness were significantly different in those with BFRBs compared to controls. Having shown the large effect size association with neuroticism, the question remain as to what exactly is the relationship between neuroticism and these disorders? Research meta-analysis has shown that neuroticism seems to be a trait associated with multiple psychiatric disorders (albeit stronger in some than others, see [27, 28]), with the effect size seen here on par with that reported in mood and anxiety disorders and higher than that reported in substance use disorders [29]. The lack of specificity of neuroticism to BFRBs suggest that perhaps this personality trait is a vulnerability marker to many disorders, to varying degrees, including BFRBs. Additionally, as with other disorders, the causal directionality of our findings merits future study. Does the tendency to experience negative affect lead to pulling and picking or does the BFRB behavior drive the neuroticism? Longitudinal studies are needed to better understand the temporal relationship, but this second confirmatory study highlights the potential for treatment targeting negative affect in both trichotillomania and skin picking disorder.

Another significant personality variable in BFRBs was introversion (i.e. relative lack of extraversion). Introversion measures reduced social engagement, and a tendency to focus on one’s own thoughts or feelings. Interestingly, our data indicate that introversion was significantly associated with higher skin picking severity, as well as with worse mood and higher levels of perceived stress. One interpretation of these data is that extraversion may have a sort of ameliorative or ‘protective’ effect on picking severity and may be linked to better mood and less stress in both disorders. A recent study in generalized anxiety disorder demonstrated that either cognitive behavior therapy or metacognitive therapy were both effective in increasing extraversion [30]. It may therefore be worthwhile to examine whether a similar therapy could increase extraversion and possibly improve symptoms simultaneously in people with BFRBs.

Lack of conscientiousness was significantly associated with more depressive symptoms, more impulsivity, and higher perceived stress in both trichotillomania and skin picking. Conscientiousness measures a tendency to be responsible and goal-directed. The link between lack of conscientiousness and heightened impulsivity is in keeping with prior conceptualizations linking the two concepts. Recent data demonstrate that there appears to be a subtype of both trichotillomania and skin picking disorder characterized by marked impulsivity [31]. This has been further confirmed by several studies finding deficiencies on objective cognitive measures of response inhibition as well [32]. Perhaps one means of improving impulsive responding, and thereby possibly improve BFRB symptoms, would be to increase conscientiousness. A recent study in adults with depression found that mindfulness-based cognitive therapy successfully improved conscientious, albeit with small effect size [33].

Several limitations should be considered in this study. Because of the relatively small sample sizes involved, our between-group comparisons focused on all BFRBs versus controls, rather than examining all subpopulations. For the same reason, the study did not undertake multiple comparison corrections for the between-group analysis, nor did it undertake multivariate modelling, both of which would have necessitated a considerably larger sample size. Future work using larger sample sizes is needed to further confirm and extend upon these findings, including exploring the role of comorbidities.

In conclusion, this is one of very few studies to have examined personality in the context of BFRBs. We identified a number of personality features associated with BFRBs: neuroticism with very large effect size, and introversion plus lack of conscientiousness with medium effect sizes. In turn, these personality features showed a number of associations with features of illness in those with BFRBs including perceived stress, mood/anxiety, disability/impairment, and impulsivity. Some of these associations were of large effect size. The findings have potential implications for how BFRBs are assessed, as well as in terms of potential enhancement of treatment options for these neglected but prevalent conditions.

## Data Availability

The datasets generated and/or analysed during the current study are not publicly available due to confidentiality but are available from the corresponding author on reasonable request.

## Abbreviations

BIS: Barratt Impulsivity Scale (BIS-11)
MGH-HPS: Massachusetts General Hospital Hair Pulling Scale
Perceived Stress: Perceived Stress Scale
SPS-R: Skin Picking Scale-Revised
SSRT: Stop signal reaction time
SDS: Sheehan Disability Scale total score
IED: Intra-Dimensional/Extra-Dimensional Set-Shift task total errors
QOLI: Quality of Life Inventory t-score
MFQ: Mood and feeling questionnaire
FMPS: Frost Multidimensional Perfectionism Scale
CGT_RISK_ADJ: Cambridge Gamble Task risk adjustment
CGT_QUAL_DEC_MKG: Cambridge Gamble Task quality of decision-making
CGT_OVERALL_PROP_BET: Cambridge Gamble task overall proportion of points bet
SCARED: Screen for Child Anxiety and Related Disorders Adult version total score
